# Polysomnographic study in pediatric neurofibromatosis type 1

**DOI:** 10.3389/fneur.2023.1213430

**Published:** 2023-07-18

**Authors:** Marco Carotenuto, Giovanni Messina, Maria Esposito, Claudia Santoro, Diego Iacono, Karen Spruyt

**Affiliations:** ^1^Sleep Lab for Developmental Age, Clinic of Child and Adolescent Neuropsychiatry, Department of Mental and Physical Health and Preventive Medicine, Child and Adolescent Neuropsychiatry Clinic, University of Campania "Luigi Vanvitelli", Naples, Italy; ^2^Department of Clinical and Experimental Medicine, University of Foggia, Foggia, Italy; ^3^Department of Women's and Children's Health, and General and Specialized Surgery, University of Campania "Luigi Vanvitelli", Naples, Italy; ^4^Neuropathology Research, Biomedical Research Institute of New Jersey, BRInj, Cedar Knolls, NJ, United States; ^5^Department of Pediatrics, Neuropathology Research, Mid-Atlantic Neonatology Associates (MANA), Atlantic Health System (AHS), Morristown, NJ, United States; ^6^NeuroDiderot INSERM, Université de Paris, Paris, France

**Keywords:** pediatric neurofibromatosis type 1, sleep, polysomnography, sleep duration, rare disease

## Abstract

**Background:**

Neurofibromatosis type 1 (NF1) is a genetic disease that alters neurodevelopment. We aimed to analyze the sleep macrostructure of a sample of children affected by NF1 without neurocognitive co-morbidities and MRI reports of unidentified bright objects (UBOs).

**Methods:**

A 100 pre-pubertal children participated in the cross-sectional study: 50 subjects were children diagnosed with NF1 and 50 subjects were typically developing healthy children (TDC). All participants underwent polysomnographic evaluation through which conventional sleep parameters were collected: Total sleep time (TST), Sleep latency (SOL), first REM latency (FRL), number of stage shifts/h (SS/h), number of awakenings/h (AWN/h), wake after sleep onset (WASO%), sleep efficiency percentage (SE%), percentage of sleep time spent in sleep stages 1 (N1%) and 2 (N2%), slow-wave sleep (N3%), and REM sleep (REM%). Additionally, nocturnal respiratory events such as apnea/hypopnea index (AHI), oxygen desaturation index (ODI), and periodic limb movement index (PLMI) were recorded.

**Results:**

Neurofibromatosis type 1 children showed a reduction in sleep duration parameters (TST; *p* < 0.001), sleep efficiency (SE%; *p* < 0.001), and stage N2% (*p* < 0.001). Moreover, the number of awakenings per hour (AWN/h), wake after sleep onset (WASO%), and respiratory events such as AHI, ODI, and PLMI resulted higher in NF1 vs. TDC children.

**Conclusion:**

The data showed that the sleep macrostructure differs between NF1 and TDC children. These findings suggest that the evaluation of sleep may provide useful support in corroborating the diagnosis and offers additional therapeutic management perspectives in NF1 and genetic neurodevelopmental disorders in general.

## 1. Introduction

Neurofibromatosis type 1 (NF1; OMIM#162200) (1) is a rare disease with a worldwide incidence of ~1 in 3,000 newborns. NF1 due to heterozygous mutations of the NF1 gene and subsequent haploinsufficiency of the encoded protein product neurofibromin (1–3).

Neurofibromatosis type 1 can affect several organs and systems. Among others, the central nervous system (CNS) is frequently affected in these children with many manifestations including cognitive, behavioral, neurodevelopmental disorders, speech disorders and motor impairment (4–6), migraine or headache (7–9), cerebrovascular diseases (10, 11), brain tumors, optic pathways gliomas, cranial nerves neurofibromas, aqueductal stenosis, and cerebral high-signal lesions on T2-weighted MRI (12, 13). On the other hand, among children and adolescents affected by NF1, also multiple types of epileptic disorders can appear, which are not directly or only explained by the presence of cerebral malformation (14–16).

Among the NF1-related neurological morbidities, sleep disorders (e.g., sleep-related breathing disorder, insomnia) are very frequently seen in clinical practice. However, a limited number of studies focusing on NF1-related sleep disorders have been described (16–18).

To date, in fact, only a few data on the sleep habits of NF1 patients have been reported such as a single case report of an old man suffering from sleep apnea syndrome due to the mechanical superior vena cava obstruction (16), or two questionnaire-based pediatric studies (17, 18). Following these initial studies, starting from 2016, a polysomnographic examination has been strongly recommended and considered mandatory for airway plexiform neurofibromas in NF1 subjects (19). More specifically, in 2005, Johnson et al. (17) showed a higher prevalence of NREM parasomnias in the NF1 vs. a healthy group, whereas Licis et al. (20) reported a higher rate of sleep problems (i.e., disturbances in initiating and maintaining sleep, sleep–wake transition disorders, night awakenings, and nocturnal hyperhidrosis) in NF1 vs. control children (19). Specifically, only one group of sleep problems, parasomnias such as sleepwalking and sleep terrors, had a higher occurrence (*p* ≤ 0.05) in the NF1 group than in the general population (17). The overall prevalence rate of sleep disorders reported was 6.3%. Patients with NF1 and comorbid ADHD had a higher prevalence of sleep onset and maintenance disorders (18 vs. 6.3%), sleep–wake transition disorders (12.5 vs. 6.3%), and daytime sleepiness (12.5 vs. 7.9%); differences were not statistically significant. A statistically significant difference was found in the subdomain of nocturnal hyperhidrosis (21.9 vs. 6.3%, *p* < 0.05). Patients with NF1 and IQ < 85 showed higher prevalence rates of daytime sleepiness (20 vs. 6.7%) and of sleep hyperhidrosis (11 vs. 0%).

Moreover, the sleep disturbance rate among children with NF1 has been estimated to range from 13 to 86% vs. 11–37% among typical control children (21–23).

To the best of our knowledge, polysomnographic evaluation reports in pediatric NF1 have been extremely rare, with a lack of reference data to guide clinical practice in the management of sleep problems in children and adolescents with NF1.

We aimed to systematically assess sleep macrostructure, nocturnal respiratory events, and periodic limb movements index in a sample of children affected by NF1 in comparison to TCD.

## 2. Materials and methods

### 2.1. Ethics approval

The clinical retrospective study was conducted according to the principles of the Declaration of Helsinki (24). The Ethics Committee at the Università degli Studi della Campania “Luigi Vanvitelli” approved the retrospective study design and all procedures considering the adherence to international guidelines (Protocol number 0015908/i; May 21st 2021). Participants provided informed consent and assent in the case of minors.

### 2.2. Study design

The present experimental design consisted of a retrospective cross-sectional analysis between two independent groups of subjects. NF1 subjects were recruited during the period 2003–2013 and compared with a historical control group of typical developing healthy children (TDC).

### 2.3. Study population

The NF1 subjects group consisted of 50 pre-pubertal children (27 males and 23 females; mean age 9.12 ± 1.86 years), which underwent overnight full polysomnography (PSG) after at least one night of adaptation to avoid the first night effect accordingly to international criteria for PSG (18) (Supplementary Figure 1).

The diagnosis of NF1 for all subjects was based on the National Institute of Neurological Disorders and Stroke (NIH) criteria (25).

The second group, the control group, consisted of 50 typically developing children (TDC; 22 males and 28 females; mean age 8.94 ± 1.52 years), which were all part of a historical control group collected among inpatient subjects, admitted for assessment of recurrent episodes of headache and abdominal pain, that resulted negative for neuropsychiatric disorders and pediatric screenings during hospitalization.

All subjects enrolled in this study were Caucasian, Italian native speakers, and comparable for socio-economic and educational status.

We considered as exclusion criteria for all participants: obesity and overweight, epilepsy, psychiatric disorders (i.e., autism spectrum disorders, ADHD, psychosis), cognitive disability (Intelligent Quotient assessed with the Wechsler Intelligence Scale for Children-IV <70), plexiform neurofibromas involving head or neck, psychotropic drugs treatment and MRI report of unidentified bright objects (*UBOs*).

### 2.4. Polysomnographic sleep recordings

#### 2.4.1. Sleep stage scoring

As reported in Roccella et al. (26), sleep macrostructure, nocturnal respiratory events per hour (apnea/hypopnea index, AHI; oxygen desaturation index, ODI), and periodic limb movement index (PLMI) were visually scored according to international standard criteria for pediatric age (27–30).

Specifically, the following conventional sleep parameters were evaluated by an expert scorer (MC): Total sleep time (TST), Sleep latency (SOL), first REM latency (FRL), Number of stage shifts/h (SS/h), Number of awakenings/h (AWN/h), Sleep efficiency (SE%), Percentage of sleep time spent in sleep stages 1 (N1%) and 2 (N2%), slow-wave sleep (N3%), and REM sleep (REM%).

All variables were analyzed by Hypnolab 1.2 sleep software analysis (SWS Soft, Troina, Italy). All full overnight PSGs were recorded with 19 electrodes (Fp1, Fp2, F3, F4, C3, C4, T3, T4, P3, P4, T5, T6, O1, O2, Fpz, Fz, Cz, Pz, and Oz) referenced to the contralateral mastoid (A1 and A2), left and right electrooculogram (ROC and LOC), chin electromyogram (EMG), left and right tibialis EMG, electrocardiogram (one derivation), nasal cannula, thorax and abdominal effort, peripheral oxygen saturation, and pulse and position sensors.

Respiratory nocturnal events were identified according to the criteria of the American Academy of Sleep Medicine (31) and the apnea–hypopnea index (AHI) was defined as the average number of apneas and/or hypopneas per hour. Therefore, the diagnosis of OSAS has been considered the cut-off value of AHI > 1 and the presence at least of snoring or nocturnal labored breathing or referred diurnal sleepiness (32).

For the assessment of limb movement events (PLMs), a PLMs index (number of PLMs per hour of sleep) higher than ≥5 was considered clinically significant (31).

### 2.5. Statistical analysis

Assumption of normality was performed with Kolmogorov–Smirnov test. Considering the violation of the normal distribution, descriptive statistics were expressed as medians and interquartile ranges (IQR) for continuous variables. Comparisons of categorical data were performed with the chi-squared test and Fisher’s exact test, while continuous data were analyzed with the nonparametric Mann–Whitney U test (*U*). Cohen’s *d* was calculated to establish clinical differences. A value of *p* ≤ 0.05 was considered statistically significant. The software Statistica version 8.1 (StatSoft Inc., Tulsa, OK, United States) was used for all statistical tests.

## 3. Results

The two groups did not differ in gender, age, and BMI *z*-score. However, a difference was found for IQ (Table 1), with none of the NF1 subjects having an IQ ≥ 85. None of the participants used drugs.

**Table 1:** Main characteristics of neurofibromatosis type 1 (NF1) and typical developing healthy children (TDC).

	NF1 (*n* = 50)	TDC (*n* = 50)	Test-statistic	*p*
Age	9.08 ± 1.27	9.06 ± 1.30	*U* = 0.077	0.938
Gender (Males/Females)	27/23	22/28	χ^2^(1) = 0.64	0.424
BMI *z*-score	0.62 ± 0.33	0.52 ± 0.31	*U* = 1.538	0.127
IQ	78.18 ± 2.47	99.14 ± 4	*U* = -31.519	<0.001

Table 2 summarizes descriptive values of nocturnal sleep macrostructural parameters registered in NF1 and TDC children and their statistical differences. Specifically, NF1 subjects showed a reduction in sleep duration (TST; *p* < 0.001), sleep efficiency percentage (SE%; *p* < 0.001), and N2 percentage (*p* < 0.001). Moreover, we found that NF1 children had an increase in the number (AWN/h; *p* < 0.001) and duration of wake periods (WASO%; *p* < 0.001) in comparison to TDC. Furthermore, respiratory events such as AHI (*p* < 0.001), ODI (*p* < 0.001), and PLMI (*p* < 0.001) were higher in NF1 vs. TDC group.

**Table 2:** Comparison of nocturnal sleep macrostructural parameters between neurofibromatosis type 1 (NF1) and typical developing control children (TDC).

	NF1 (*n* = 50)		TDC (*n* = 50)		M-W U		Cohen’s *d*	
	Median	IQR	Median	IQR	*z* value	*p* value		*p* value
TST-min	404.50	335–425	534.18	491–560	−7.44	0.000	−2.3 ± 1.8	<0.001
SOL-min	23.56	5.65–58.733	21.29	8.5–28.5	−1.15	0.25	0.2 ± 1.4	0.36
FRL-min	90.50	90.5–125	125.88	86–150	1.74	0.08	−0.3 ± 1.4	0.20
SS-h	9.40	6.2–10.7	8.34	4.8–10.9	−1.41	NS	0.1 ± 1.4	0.61
AWN-h	7.00	4.9–8.7	1.914	0.3–3.1	−5.43	0.000	1.9 ± 1.7	<0.001
SE%	80.03	71.93–87.55	90.652	87.9–93.6	−2.81	0.005	−1.2 ± 1.5	<0.001
WASO-%	16.44	12.11–17.32	4.508	0.6–7.4	−5.43	0.000	2.1 ± 1.8	<0.001
N1-%	3.01	2.31–3.74	2.822	0.7–3.5	−3.22	0.001	0.4 ± 1.4	0.08
N2-%	29.15	21.85–31.1	41.518	35.2–45.9	−6.43	0.000	−1.2 ± 1.5	<0.001
N3-%	28.12	26.73–33.33	29.908	23.8–35.6	−1.01	NS	0.6 ± 1.4	0.003
REM-%	22.67	20.44–26.55	21.228	16.3–26	−1.61	NS	0.8 ± 1.5	<0.001
AHI	5.94	3.44–9.4	0.882	0.7–1.1	−9.85	0.000	1.9 ± 1.7	<0.001
ODI	2.31	0.9–4.8	0.38	0.2–0.5	−5.43	0.000	1.3 ± 1.6	<0.001
PLMI	5.35	3.62–9.5	3.394	2.7–3.9	−7.24	0.000	1.0 ± 1.5	<0.001

TST, total sleep time; SOL, sleep onset latency; FRL, first REM sleep latency; SS, stage shifts; AWN, awakenings; SE, sleep efficiency%; WASO, wake time after sleep onset; N1, sleep stage 1; N2, sleep stage 2; N3, slow-wave sleep; REM, rapid eye movement sleep, AHI, apnea/hypopnea index/h; ODI, oxygen desaturation index percentage; PLMI, periodic limbs movement index.

Values of *p* < 0.05 were considered significant.

IQR, inter-quartile range.

M-W U, Mann–Whitney U test.

Children with NF1 have severe sleep-disordered breathing, i.e., 6.9 ± 4.5 AHI, with all having an AHI > 1. 42% of the control children have an AHI > 1. None of the control children had a PLMI≥5 but 56% of NF1 had. While 66.67% of the NF1 children had an AHI > 5 and PLMI ≥ 5.

*Post hoc* comparisons between the clinical groups within the NF1 sample showed that clinical groups were different for N2%. Particularly, those with periodic leg movements but moderate sleep-disordered breathing spent more time in N2% compared to those with severe sleep-disordered breathing. IQ (*p* = 0.5019) and BMI *z*-score (*p* = 0.7375) were not different across these clinical groups (Table 3).

**Table 3:** Comparison of nocturnal sleep macrostructural parameters between clinical groups of NF1 children.

	(1) AHI > 1 and PLMI < 5	(2) AHI > 5 and PLMI < 5	(3) AHI > 1 and PLMI ≥ 5	(4) AHI > 5 and PLMI ≥ 5	Kruskal-Wallis test: H(3, *n* = 50)	*p* value
TST-min	386.2 ± 54.9	349.8 ± 53.1	407.7 ± 29.2	370.4 ± 83	3.80	0.28
SOL-min	14.1 ± 51	46.5 ± 62.6	9.4 ± 45.8	37.5 ± 62.7	3.60	0.31
FRL-min	103.6 ± 19.4	147.9 ± 88.9	89.8 ± 6.8	110.7 ± 36.1	6.84	0.08
SS-h	8.6 ± 2.5	10.8 ± 3	7.5 ± 2.3	8.2 ± 3.1	5.36	0.15
AWN-h	5.9 ± 2.4	8.4 ± 4.4	6.6 ± 2.4	7.3 ± 3.2	2.13	0.55
SE%	83 ± 12.2	77.2 ± 10.6	82.9 ± 6.9	77.1 ± 13.5	2.34	0.51
WASO-%	67.3 ± 31.9	58 ± 25.5	76.1 ± 19	70.8 ± 25	1.31	0.72
N1-%	5.7 ± 5.2	4.6 ± 3.6	3.7 ± 0.3	3.7 ± 2.2	1.15	0.76
N2-%	30.6 ± 8.3	32 ± 14.8	37.1 ± 1.2	29.9 ± 12	8.60	0.04
N3-%	38.7 ± 12	37.3 ± 6.7	33.4 ± 2.5	36.9 ± 6.9	1.50	0.68
REM-%	25.1 ± 5.2	26.1 ± 11.3	25.7 ± 3.4	29.5 ± 6.5	6.12	0.11
ODI	1.7 ± 1.7	1.9 ± 1.8	4 ± 4.1	4.8 ± 3.6	10.81	0.01
IQ	77.9 ± 2.3	78 ± 2.7	77.5 ± 2.3	78.7 ± 2.6	2.36	0.50
BMI *z*-score	0.5 ± 0.3	0.6 ± 0.3	0.6 ± 0.2	0.7 ± 0.4	1.26	0.74

Values of *p* < 0.05 were considered significant.

## 4. Discussion

This is the first study describing the macrostructure of a sample of pre-pubertal children with NF1. Compared to a control group, they showed a shorter sleep duration with an increased number and length of awakenings during the sleep period. Children with NF1 further demonstrated reduced N2%. Overall, disruptions of sleep continuity may inflate the daytime neurocognitive deficits exhibited by children with NF1. While pathological brain characteristics, such as diffuse white matter lesions, have been suggested as neuronal substrates of their cognitive deficits, the adverse impact of an undiagnosed sleep disorder should not be overlooked.

Children with different neurodevelopmental disorders show a higher rate of sleep disorders than healthy comparisons (33), this is particularly true in genetic syndromes (34). Previous studies were predominantly based on parental referring and/or questionnaire-based reports and did not derive from quantitative polysomnographic data. In general, NF1-PSG reports are still rarely performed both in adult and children populations despite recurrent sleep complaints.

In our study, the NF1 group showed an increase in sleep fragmentation parameters, being increased stage shifting and awakenings per hour, as well as a lower percentage in sleep stage N2. Next, respiratory parameters and PLMI in NF1 children were in clinical ranges. That is, increased AHI and ODI, as well as PLMI, were found, suggestive of sleep disorders. These data in NF1 children have not been reported in clinical literature since most if not all, reports on nocturnal respiratory events seem to be limited to adult subjects (19).

It is important to emphasize that sleep disorders such as sleep-disordered breathing (SDB) and PLMS might determine the worsening of already poor attentional and executive functioning as well as learning difficulties in NF1 children (35, 36). Namely, SDB has been commonly associated with scholastic underperformance (37, 38) and executive dysfunction (39). Whereas PLMS were found in children with SDB (40) and attention-deficit/hyperactivity disorder (ADHD) (41, 42). The prevalence of ADHD is important and can affect the overall neuropsychological performance of children with NF1. A better definition of sleep disturbances in the NF1 patient population, in particular during childhood, could address a personalized treatment and so reduce their possible related impact on their neurocognitive deficits.

Most of the previous studies in children with NF1 have been based on questionnaires. Reporting alterations in disturbances of initiating and maintaining sleep, arousal, sleep–wake transition, and hyperhidrosis, but not reported abnormal sleep breathing or excessive daytime somnolence (19, 24). Our PSG study confirmed alterations in all sleep duration parameters suggestive of poor-quality sleep, which may lead to increased daytime sleepiness. However, this complaint is underreported or not queried. Reduced sleep duration findings, however, are coherent with some animal studies in rodents (43). A potential dysregulation of the NREM/REM cycling might be hypothesized. This hypothesis may be indeed supported by our data showing higher stage shifting per hour and rate of behavioral nocturnal awakenings percentage as a putative effect of the alteration of sleep continuity regulation.

In our NF1 subjects, the sleep stage N2% representation is decreased compared to controls. This finding might be one of the sources of the neurophysiological basis for the learning disorders and motor impairment frequently encountered in NF1 children (44, 45). Moreover, murine models of NF1 showed a dopamine deficit. This metabolic deficit could have actually, a major role in sleep balancing or regulation phenomena (46). In fact, it has been established that there is a mesolimbic dopamine pathway in sleep/wake regulation between the ventral tegmental area (VTA) and the *nucleus accumbens* (NAc) (47–49).

Finally, sleep-related breathing disorders have been rarely reported in questionnaire-based studies, differently from nocturnal hyperhidrosis, which seems to have a high prevalence among NF1 children despite that this nocturnal sign is strongly associated with nocturnal respiratory disorders (24, 50). These conflicting results may be explained by the parents’ difficulty in properly recognizing SBD contrary to PSG recording (51, 52). In fact, the alterations of nocturnal respiratory parameters might be explained by the loss of modulation of the hypothalamic–pituitary axis by defective neurofibromin (53).

In general, our findings are in line with some animal studies. In fact, experimental data about abnormalities of the circadian clock control in Drosophila models of NF1 (45) and on the defects in the regulation of RAS activity, cAMP generation, and dopamine homeostasis have been proposed as key mechanisms for sleep disruption in NF1 children (54). In addition, dopamine alterations may result in attentional and learning deficits (54–56), which could be explained by the reduced functional connectivity between the striatum and the frontoparietal networks and increased striatal functional connectivity with the limbic network in NF1 children (57). Specifically, the dopaminergic pathway alteration can indeed cause the higher PLMI in NF1 reported in our study considering that periodic limb movement disorder and the restless leg syndrome tend usually to beneficiate from dopaminergic receptor agonists treatment (58, 59) or iron supplementation in developmental age (60) due to functional interactions among iron, epicidin, and dopamine production (18).

We moreover would like to specify that the choice of recruiting pre-pubertal children with NF1 has been made to avoid sleep alterations due to hormonal abnormalities during adolescence (34, 61).

The limited availability of children to undergo PSG is mainly due to the necessity of hospitalization. This limitation joined to the relative rarity of NF1, seems to well explain the relatively small size of the recruited population. Another limitation of the present study may be identified in the PSG evaluation restricted to the children with NF1 without UBOs as well as the retrospective nature of our investigation. Lastly, the control group is a presumed healthy group without any medical diagnosis fitting the exclusion criteria.

Notwithstanding these limitations, the present study may be considered the first investigation reporting PSG data in pre-pubertal NF1 children. We confirmed some previous findings, however, we added knowledge on a series of new aspects of sleep disorders in NF1 children, specifically in NF1 children without any other neurocognitive comorbidity (53). Considering that sleep problems may impact most if not all, aspects of daily life in general and during pediatric age in particular (62), our study warrants considering a co-occurring clinical sleep disorder upon embarking on a treatment approach in children with NF1. Our findings should be interpreted in relevance to both clinical and physiopathological perspectives regards deficits shown by children with NF1. Therefore, this study confirms that PSG is needed for a detailed and objective assessment of sleep in NF1 and likely other neurodevelopmental disorders. Finally, future studies should also evaluate the effects of sleep disturbance on the neurocognitive profile in children with NF1 as well as in other genetic disorders.

## Data availability statement

The datasets presented in this article are not readily available because of ethical and privacy restrictions. Requests to access the datasets should be directed to MC, marco.carotenuto@unicampania.it.

## Ethics statement

The studies involving human participants were reviewed and approved by the Ethics Committee at the Università degli Studi della Campania “Luigi Vanvitelli” Protocol number 0015908/i; May 21, 2021. Written informed consent to participate in this study was provided by the participants’ legal guardian/next of kin.

## Author contributions

MC and KS: conceptualization and draft preparation. GM, ME, and CS: data collection. DI: statistical analysis and data elaboration. KS: paper final revision. All authors contributed to the article and approved the submitted version.

## Conflict of interest

The authors declare that the research was conducted in the absence of any commercial or financial relationships that could be construed as a potential conflict of interest.

## Publisher’s note

All claims expressed in this article are solely those of the authors and do not necessarily represent those of their affiliated organizations, or those of the publisher, the editors and the reviewers. Any product that may be evaluated in this article, or claim that may be made by its manufacturer, is not guaranteed or endorsed by the publisher.
