# Supplementary figures and images for: Polysomnographic study in pediatric neurofibromatosis type 1
Source: Front Neurol. 2023 Jul 18;14:1213430. doi: 10.3389/fneur.2023.1213430 (PMC10394094; doi:10.3389/fneur.2023.1213430)

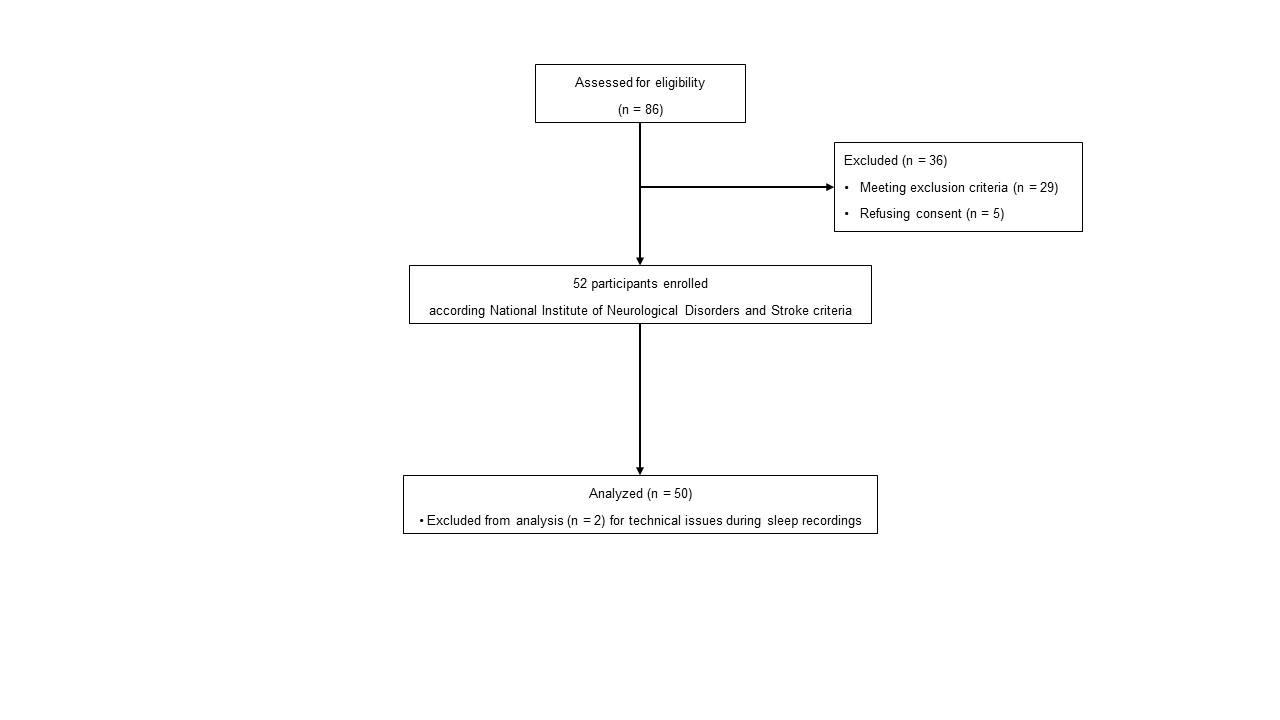

Supplement: Supplementary file 1 [file Figure_1.JPEG]
